# Contemporary treatment trends for upper urinary tract stones in a total population analysis in Germany from 2006 to 2019: will shock wave lithotripsy become extinct?

**DOI:** 10.1007/s00345-021-03818-y

**Published:** 2021-08-28

**Authors:** Roman Herout, Martin Baunacke, Christer Groeben, Cem Aksoy, Björn Volkmer, Marcel Schmidt, Nicole Eisenmenger, Rainer Koch, Sven Oehlschläger, Christian Thomas, Johannes Huber

**Affiliations:** 1 Department of Urology, University Hospital Carl Gustav Carus, TU Dresden, Fetscherstraße 74, 01307 Dresden, Germany; 2 Department of Urology, Klinikum Kassel, Kassel, Germany; 3 Coloplast GmbH, Hamburg, Germany; 4 Reimbursement Institute, Hürth, Germany

**Keywords:** Urolithiasis, Percutaneous nephrolithotomy, Ureterorenoscopy, Extracorporeal shock wave lithotripsy, Health services research

## Abstract

**Purpose:**

To describe the change in upper urinary tract stone management in Germany over a 14-year period.

**Methods:**

Using remote data processing we analyzed the nationwide German billing data from 2006 to 2019. To analyze the clinics’ case numbers and regional trends, we used the reimbursement.INFO tool based on standardized quality reports of all German hospitals. To also cover shock wave lithotripsy (SWL) as an outpatient procedure, we analyzed the research database of the Institute for Applied Health Research with a representative anonymous sample of 4 million insured persons.

**Results:**

The number of inpatient interventional therapies for upper tract urolithiasis in Germany increased from 70,099 cases in 2006 to 94,815 cases in 2019 (trend *p* < 0.0001). In-hospital SWL declined from 41,687 cases in 2006 to 10,724 cases in 2019 (decline of 74%; trend *p* < 0.0001). The percentage of SWL as an outpatient procedure increased between 2013 and 2018 from 36 to 46% of all performed SWL, while total SWL case numbers declined. Contrarily, the number of ureteroscopies increased from 32,203 cases in 2006 to 78,125 cases in 2019 (increase of 143%; trend *p* < 0.0001). The number of percutaneous nephrolithotomy also increased from 1673 cases in 2006 to 8937 in 2019 (increase of 434%; trend *p* < 0.0001).

**Conclusion:**

We observed an increase in interventional therapy for upper tract urolithiasis in Germany with a dramatic shift from SWL to endoscopic/percutaneous treatment. These changes may be attributed to enormous technological advances of the endoscopic armamentarium and to reimbursement issues.

**Supplementary Information:**

The online version contains supplementary material available at 10.1007/s00345-021-03818-y.

## Introduction

Urolithiasis is a worldwide health issue with millions of people affected by symptomatic calculi in the upper urinary tract every year. The most recent data on incidence and prevalence in Germany were published for the year 2001 with 1.5% and 4.8%, respectively [1]. Factors such as stone location, calculus size, anatomy of the collecting system, patient comorbidities and preference as well as surgeon preference have impact on the selection of the therapeutic modality [2]. Due to these various aspects that need to be considered, modern stone therapy needs to be individually tailored to the patient. Guidelines on urolithiasis, such as the European (EAU), the American (AUA) and the German (DGU), help in the decision-making process. However, for most urinary stones several options are available and need to be discussed with the patient [2–4]. Technological advances with miniaturization of instruments, the ability to fragment larger stones with modern lasers and the broad availability of flexible ureteroscopes have caused a shift from shock wave lithotripsy (SWL) to endoscopic or percutaneous approaches in the treatment of urolithiasis worldwide [5]. The numbers of SWL as an inpatient procedure in German hospitals were declining from 2005 to 2013 [6]. This trend has also been shown in a recently published nationwide survey in which 199 urology departments in Germany were asked about their treatment preference for urolithiasis. In summary, 87% of urological departments stated that less than 30% of all urinary stones are treated with SWL. 43% of all departments stated that 51–70% of all stone therapy is done via ureterorenoscopy (URS) and further 43% of all departments stated that more than 70% of all cases are managed via URS [7].

The aim of this study was to evaluate treatment trends for upper urinary tract stones in Germany over a study period of 14 years.

## Materials and methods

We followed the "REporting of studies Conducted using Observational Routinely collected health Data" (RECORD) statement [8].

### Nationwide billing data in Germany (diagnosis-related groups database)

A total population analysis of the nationwide billing data in Germany from 2006 to 2019 was performed. In 2004 the international diagnosis related groups (DRG) had been implemented in the German health care system, thus regulating the reimbursement of inpatient treatment. The DRG are comprised of the diagnosis coded via the ICD-10 (International Statistical Classification of Diseases and Related Health Problems) code and an OPS code (German adaption of the International Classification of Procedures in Medicine) for the performed intervention. Data of each treated case are first transferred to the Institute of Hospital Remuneration and subsequently to the German Federal Statistical Office (Destatis). The nationwide Destatis database contains every reimbursed inpatient case in Germany except for psychiatric, forensic, and military hospitals. We previously described this methodology in more detail [9, 10].

For data analysis we combined a specific code for urolithiasis as primary or secondary diagnosis (Table S1) with a specific OPS-code (Table S2). SWL and percutaneous nephrolithotomy (PCNL) have very specific OPS codes, which are exclusively used for urolithiasis. That is not true for ureteroscopy. However, by combining a urolithiasis-specific ICD code with a specific OPS code we were able to identify procedures that were performed for upper tract calculi. Also, in 2010 a specific OPS code for flexible ureteroscopy was introduced, which gave us the opportunity to discriminate between semirigid and flexible ureteroscopy for the years 2010–2019.

### InGef research database

URS and PCNL are not routinely performed in an outpatient setting in Germany. In contrast, SWL is performed as an inpatient or outpatient procedure varying between hospitals and regions within Germany. To also cover SWL as an outpatient procedure, we analyzed the research database of the Institute for Applied Health Research (InGef). For this purpose, we extracted the ratio of inpatient to outpatient SWL for every year from 2013 to 2018 and used this ratio to estimate the number of outpatient SWL treatments per year from the known number of inpatient procedures from the Destatis database. The InGef research database is an anonymized claims database with high external validity, comprising longitudinal data from approximately 6.7 million persons insured in one of 70 German statutory health insurances [11]. For the purpose of this analysis, the InGef Research Database was condensed to a sample of approximately 4 million insured people which is representative of the German population in terms of age and region [11].

### Hospital quality reports

Hospitals in Germany are obliged to record and report data concerning diagnosis and treatment annually (ICD and OPS codes). We used the analysis tool “reimbursement.INFO” (RI Innovation GmbH, Hürth, Germany) to extract data on hospital procedures for treating upper urinary tract stones for the years 2006 to 2019. For small annual caseloads of 1, 2 or 3 cases the exact number was not provided, but instead 1 for data protection reasons. The maps were rendered with the software easymap^©^ office (Lutum + Tappert DV-Beratung GmbH, Bonn, Germany).

### Data protection and ethics statement

We performed all actions in accordance with the Declaration of Helsinki in its latest version. Analyzed data were completely anonymized and derived from established databases with rigorous data protection measures. Therefore, an additional ethics statement was not required.

### Statistics

Linear regression for trend analysis and Wald tests were performed with SAS V9.4 (SAS Institute, Cary, NC).

## Results

Unless otherwise noted, all trends refer to the total study period from 2006 to 2019.

**Fig. 1:**
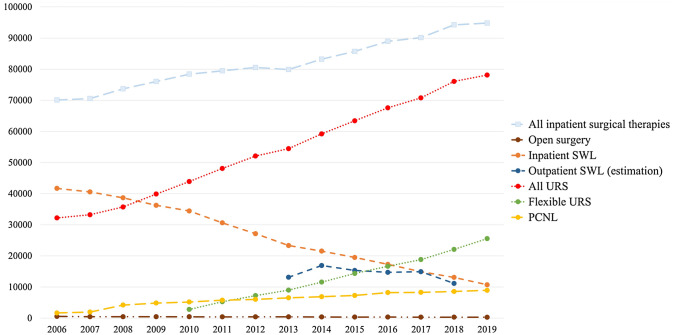
Surgical therapies for upper tract urinary calculi in Germany from 2006 to 2019

We observed an increasing case number of surgically treated upper tract urinary stones from 70,099 to 94,815 cases (total increase of 35% or 2% per year; trend *p* < 0.0001). The number of clinics performing these therapies declined from 512 to 481 (trend *p* < 0.0001). The mean patient age at intervention was 53 ± 15.7 years and 68% were males. Both, age and the sex ratio did not significantly change over time. The mean LOS for all interventions considerably decreased from 4.7 ± 4.6 days to 3.4 ± 4 days (trend *p* < 0.0001). Figure 1 provides an overview of all surgical therapies for upper tract urinary calculi in Germany from 2006 to 2019.

**Fig. 2:**
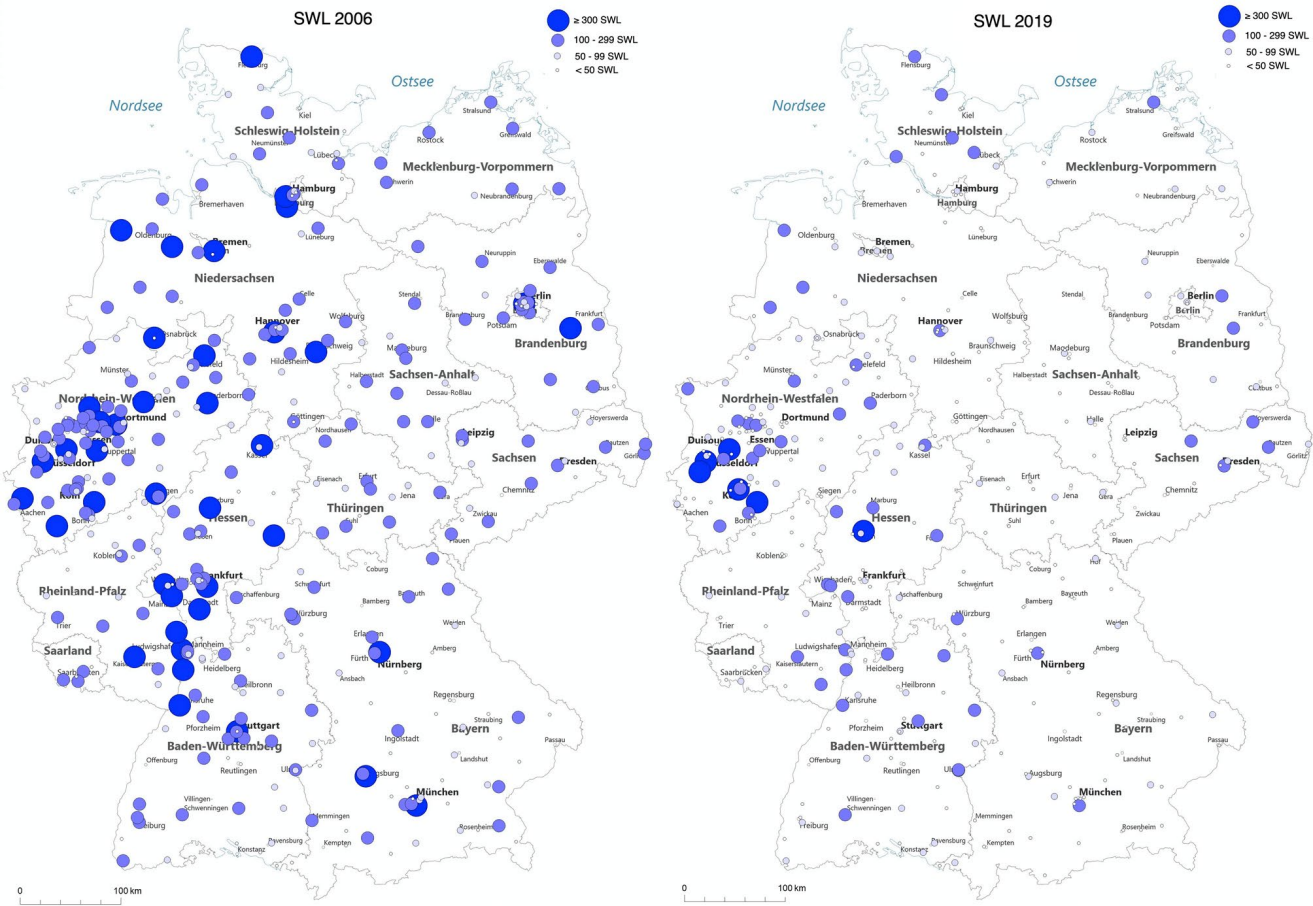
Regional changes in SWL case numbers in Germany from 2006 to 2019

We observed a steady decline in inpatient SWL from 41,687 cases in 411 clinics to 10,724 cases in 325 clinics, i.e. a total decline of 73% or 10% per year (trend *p* < 0.0001). This SWL decline is mirrored by the regional changes displayed in Fig. 2. 77% of SWL were performed for kidney stones and 23% for ureteral stones. The mean LOS decreased from 4.4 ± 4.4 days to 3.1 ± 3.2 days (trend *p* < 0.0001). We observed a shift towards more outpatient procedures with 36% of all SWL done in an outpatient setting in 2013 versus 46% in 2018. The estimated number of outpatient SWL cases were 13,122 in 2013, 16,916 in 2014, 15,319 in 2015, 14,711 in 2016, 14,918 in 2017, and 11,152 in 2018.

The total number of URS for upper tract urolithiasis showed a steady increase from 32,203 cases to 78,125 cases with an average increase of 7% per year (total increase of 143%; trend *p* < 0.0001). Regional changes for URS (for all indications) are depicted in Figure S1. 495 hospitals in Germany reported stone-related URS in 2006 and 475 hospitals in 2019 (trend *p* = 0.0009). The mean LOS decreased over time from 5.3 ± 4.6 days to 3.1 ± 3.7 days (trend *p* < 0.0001). In 2010, 2785 flexible URS cases were recorded in 146 clinics. In the year 2019, there were 25,541 flexible URS cases performed in 356 clinics (total increase of 817%; trend *p* < 0.0001). On average we observed a 30% increase in flexible URS per year (shown in figure S2). The mean LOS decreased over time from 4.3 ± 3.6 days in 2010 to 3.1 ± 4.1 days in 2019 (*p* < 0.0001). A single-use ureteroscope was coded for 2,775 cases in 2018 and for 4,432 cases in 2019 (*p* < 0.0001).

The number of PCNL increased from 1673 cases to 8937 cases (average increase of 16% per year and a total increase of 434%; trend *p* < 0.0001). PCNL was performed in 282 hospitals in Germany in 2006, and the number steadily increased to 364 clinics in 2019 (trend *p* = 0.0002). This increase in hospitals that offer PCNL is depicted in figure S3. The mean LOS declined from 12 ± 8.2 days to 6.5 ± 6.8 days (trend *p* < 0.0001).

The number of open procedures for upper tract urolithiasis steadily declined from 553 cases in 237 clinics to 294 cases in 163 clinics, i.e. a total decline of 47% or 5% per year (trend *p* < 0.0001). The mean LOS decreased from 16.7 ± 9.5 days in 2006 to 11.2 ± 6.4 days in 2019 (trend *p *< 0.0001).

## Discussion

We present contemporary German total population data over a study period of 14 years on all surgical interventions for upper urinary tract stones. The total number of in-hospital procedures for all upper tract urolithiasis increased from 70,099 cases in 2006 to 94,815 cases in 2019 and we observed a strong trend towards endoscopic or percutaneous procedures. This increase in surgical interventions is most likely attributed to an increased life-time prevalence of urinary tract stones due to a higher prevalence of metabolic disorders in the population (obesity, metabolic syndrome, type 2 diabetes mellitus) and also, due to fact that with the widespread use of computed tomography (CT) more asymptomatic upper tract urinary calculi are being diagnosed [12, 13].

Open stone surgery is rarely performed nowadays: case numbers were low in 2006 and dropped by almost 50% over the study period. In-hospital SWL numbers were decreasing with 41,687 inpatient SWL in 2006 compared to 10,724 inpatient SWL cases in 2019 (declined by 74%).

This decline in SWL numbers was not merely caused by a shift from an inpatient to outpatient setting of the procedure, but total SWL case numbers dropped. Nevertheless, assessing SWL as an outpatient procedure in Germany is cumbersome as no single and reliable data source is available. The quality reports that German hospitals are obliged to provide, comprise all inpatient and outpatient SWL that are performed by hospitals. However, they do not account for office-based urologists with SWL machines in their office. There are no published data on how many stationary and mobile SWL machines are used by office-based urologists in Germany, but via addressing the quality reports and the InGef database, we can provide a valid estimate for the total number of outpatient SWL.

We observed a dramatic decline in the utilization of SWL, which seems to be multifactorial: First, indications for the different stone therapies have changed over the years. After its introduction in 1980, SWL was soon adopted worldwide as a minimally invasive treatment option for ureteral and kidney stones [14]. However, the advances in endoscopic instruments in the late 1990s and early 2000s, and the introduction of laser fragmentation led to a decline in utilization of SWL. Furthermore, in contrast to the advances in our endoscopic armamentarium, improvements in SWL technique have not been likewise implemented in clinical practice over the last two decades. Recent developments such as enlargement of the focal zone, enlargement of the focus, improvement of efficacy of electrohydraulic shock-wave source, “burst-SWL”, and high-frequency shock waves have not yet been addressed by the shrinking lithotripter market [15–20].

Second, there are factors specific for Germany with regards to billing and reimbursement when it comes to SWL. Acceptance of SWL as in-patient treatment by health insurance companies has become very difficult, since it can be performed safely as an outpatient procedure in most patients. However, reimbursement of outpatient SWL is on an average 66% lower than for inpatient treatments [21]. Therefore, reimbursement issues could possibly represent another driving factor in the change from SWL to endoscopic treatments. Patients that are admitted to the hospital ward for a procedure might as well undergo endoscopic treatment instead of SWL. This is even more so the case, when beforehand a ureteral stent has been established in the emergent state of a symptomatic, blocking ureteral stone. Usually, patients are discharged after a ureteral stent has been placed and the obstruction was relieved. This practice is very common in Germany and only few patients undergo an immediate “in situ” SWL or “emergency SWL” [22, 23].

Last, from our personal experience, more and more patients tend to opt for an efficient and fast stone therapy, favoring URS or PCNL as first choice compared to SWL [24].

As a result of the strong decline in SWL, we observed an increase in endoscopic upper urinary tract stone therapies. Today, modern holmium and thulium lasers can be utilized through flexible instruments and fragment any stone irrespective of its composition. Another cornerstone in modern URS was the introduction of the “dusting”-technique. Likewise, in PCNL progress has been made with miniaturization and improvements in instruments and auxiliary tools like the Holmium laser. All these aspects and the equalization of endoscopic approaches with SWL in the primary treatment of upper tract stones < 10 mm in national and international guidelines, led to a dramatic increase in the number of endoscopic treatments for upper tract stones composed of the increment in URS (+ 143%) and PCNL (+ 434%).

The German DRG reimbursement system is intended to be a “learning system” and every year adjustments are made to keep health costs under control [6]. We assume that this fact led to a downgrade of SWL with regard to reimbursement, in favor of URS and PCNL, hence impeding the acquisition of new SWL machines. We observed a significant reduction in the LOS for patients after URS from 5.3 to 3.1 days, which is one way for hospitals to compensate for the high costs. As of 2019 the LOS was the same for URS and SWL. The relatively long hospital stay for SWL patients can be explained by patients undergoing multiple SWL sessions (every other day in an inpatient setting) and by the fact that SWL is part of a DRG group of surgeries with high invasiveness (including retroperitoneal surgery) and hospitals are facing cuts in remuneration if patients are discharged too early.

With the growing PCNL experience and smaller nephroscopes (mini-PCNL) urologists started to treat smaller kidney stones via PCNL. Recently, more and more experienced centers have implemented tubeless mini-PCNL for small stones, which can result in a faster stone free state for the patient and higher reimbursement. For PCNL we observed the most dramatic decrease in length of hospital stay from 12 to 6.5 days.

Similar trends are reported for England with increasing URS and PCNL cases between 2006 and 2014 [25]. However, in contrast to our data, SWL case numbers remained stable over the study period. Additionally, in England almost 25% of URS cases were done as day-case URS, whereas same day discharge is a rarity in Germany [25].

Many countries have introduced the diagnosis-related groups (DRG) system since the early 2000s, including England, Finland, France, Germany, Poland, the Netherlands, Sweden, and Switzerland, to increase transparency and to ensure a rational and economic use of resources in the health care systems [26]. Regardless of its achievements, significant improvements and changes to the existing DRG system in Germany must be made, especially outpatient procedures and day-case surgeries should be reinforced. Certainly, the observed trends in upper urinary stone treatment in Germany cannot be extrapolated to other countries; thus efforts shall be made to gather more epidemiological data on stone therapy in various countries and health care systems.

Shared decision making with active participation of the patient in the process is of utmost importance, especially given that, according to the findings of Omar et al. up to 85% of patients are likely to defer the final decision on upper tract stone therapy to the physician [27]. Consequently, reimbursement must not be a key factor when patients are counselled for choosing their stone therapy.

Our study has several limitations: The given numbers in this work are case numbers and do not represent individual patients. Patients may have had multiple interventions and various combinations of the above-mentioned stone therapies. Furthermore, our data do not give information on comorbidity, calculus size, calculus composition, complications, and retreatment rates. Procedural coding is linked to reimbursement and, therefore, it is possible that specific codes with higher reimbursement rates are used more frequently, where applicable. Nevertheless, extracting billing data on upper tract urolithiasis has proven to yield valid results in various other studies [25, 28–30].

## Conclusion

The number of SWL performed in Germany has been steadily declining over the past decade and the worldwide trend towards endoscopic/percutaneous procedures has been confirmed in the German health system. The fate of SWL in upper urinary tract stone management will depend on the implementation of recent technological developements and on finding a suitable framework for remuneration within the German health care system.

## Supplementary Information

Below is the link to the electronic supplementary material.

Supplementary file1 (JPG 3952 KB)

Supplementary file2 (JPG 62 KB)

Supplementary file3 (JPG 3362 KB)

Supplementary file4 (DOCX 17 KB)

## Data Availability

All data used in this work are stored centrally at the specific institutes (German Federal Statistical Office – Destatis; “reimbursement.INFO”—RI Innovation GmbH; InGef Research Database).
