# Supplementary figures and images for: Contemporary treatment trends for upper urinary tract stones in a total population analysis in Germany from 2006 to 2019: will shock wave lithotripsy become extinct?
Source: World J Urol. 2021 Aug 28;40(1):185–91. doi: 10.1007/s00345-021-03818-y (PMC8813696; doi:10.1007/s00345-021-03818-y)

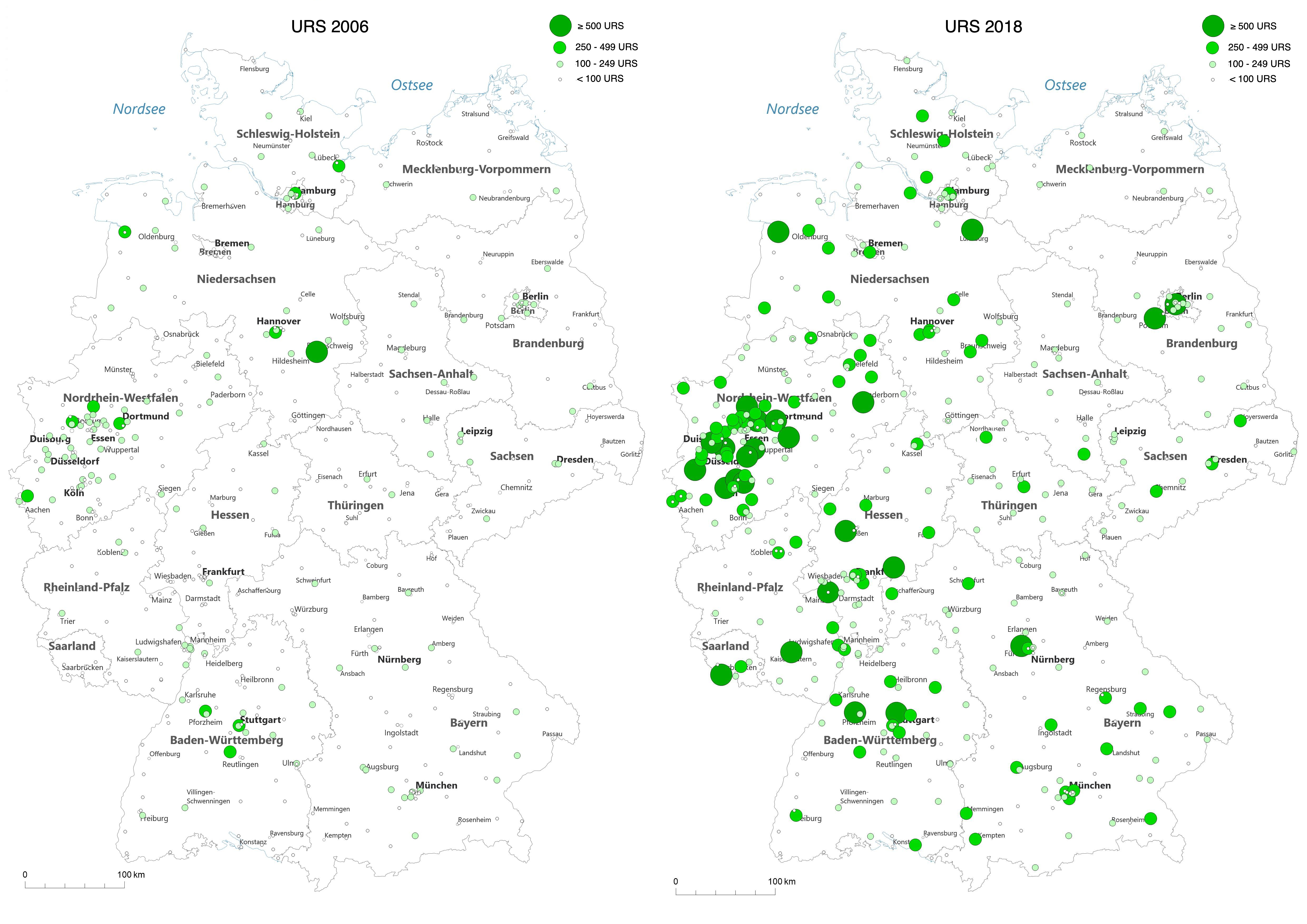

Supplement: Supplementary file 1 — Supplementary file1 (JPG 3952 KB) [file 345_2021_3818_MOESM1_ESM.jpg]

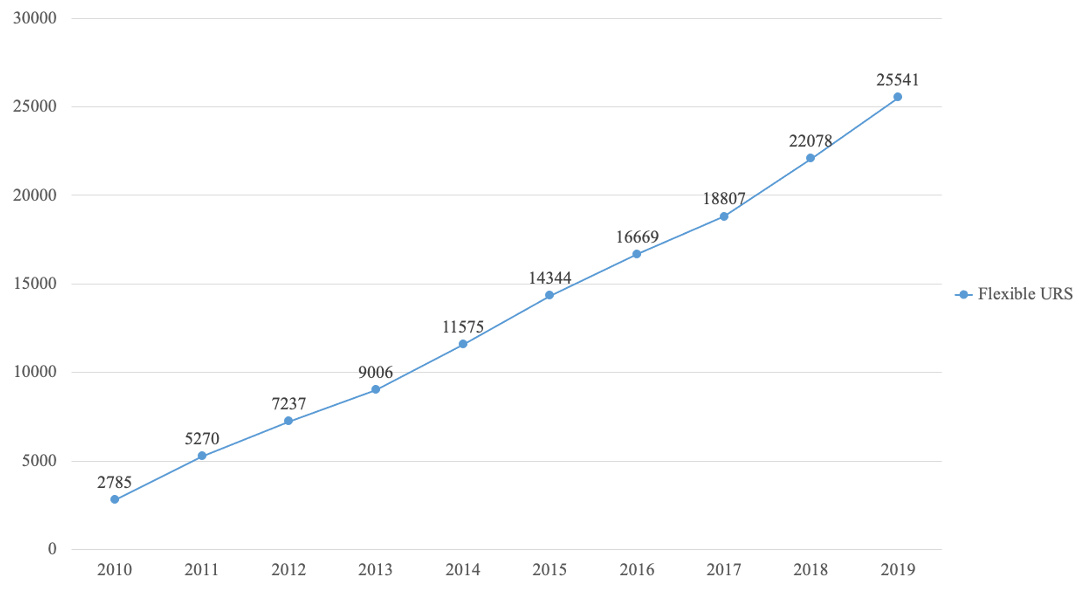

Supplement: Supplementary file 2 — Supplementary file2 (JPG 62 KB) [file 345_2021_3818_MOESM2_ESM.jpg]

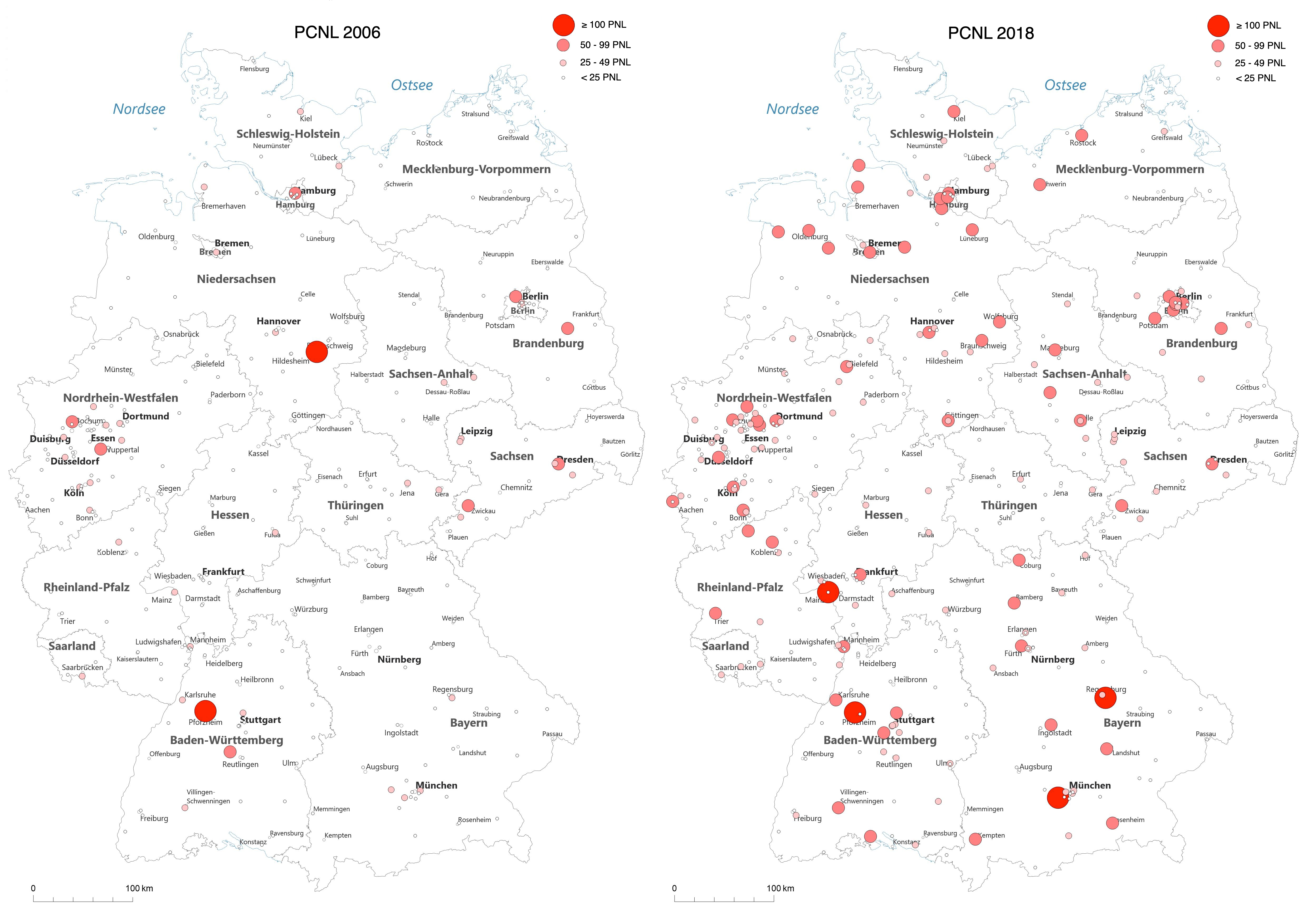

Supplement: Supplementary file 3 — Supplementary file3 (JPG 3362 KB) [file 345_2021_3818_MOESM3_ESM.jpg]
