# Supplementary material for: Contemporary treatment trends for upper urinary tract stones in a total population analysis in Germany from 2006 to 2019: will shock wave lithotripsy become extinct?
Source: World J Urol. 2021 Aug 28;40(1):185–91. doi: 10.1007/s00345-021-03818-y (PMC8813696; doi:10.1007/s00345-021-03818-y)
Supplement: Supplementary file 4 — Supplementary file4 (DOCX 17 KB) [file 345_2021_3818_MOESM4_ESM.docx]

**Supplements**

**Table S1. Urolithiasis-related ICD-10 codes**

| **Description** | **ICD-10 code** |
| --- | --- |
| Calculus of kidney (without hydronephrosis) | N20.0 |
| Calculus of ureter (without hydronephrosis) | N20.1 |
| Calculus of kidney with calculus of ureter (without hydronephrosis) | N20.2 |
| Urinary calculus, unspecified | N20.9 |
| Hydronephrosis with renal and ureteral calculous obstruction | N13.2 |

**Table S2. Description of Urolithiasis-related OPS codes**

| **Description** | **OPS code** |
| --- | --- |
| **Extracorporeal show wave lithotripsy** | |
| SWL ureteral stone | 8-110.1 |
| SWL kidney stone | 8-110.2 |
| SWL – not specified | 8-110.x & 8-110.y |
| **Ureterorenoscopy** | |
| Ureteroscopic extraction ureteral stone | 5-562.4 |
| Ureteroscopic extraction ureteral stone with lithotripsy | 5-562.5 |
| Ureteroscopic extraction ureteral stone with Dormia basket | 5-562.8 |
| (Ureteroscopic) Relocation of the stone | 5-562.9 |
| Extraction of a stone: ureteroscopically | 5-550.21 |
| Extraction of a stone with lithotripsy: ureteroscopically | 5-550.31 |
| *Available available since 2010* |  |
| Application of a flexible ureteroscope | 5-98b |
| *Code available since 2018* |  |
| Application of a flexible ureteroscope: single-use scope | 5-98b.0 |
| Application of a flexible ureteroscope | 1-999.2 |
| Application of a flexible ureteroscope: single-use scope | 1-999.20 |
| **Percutaneous Nephrolitholapaxy** | |
| *Codes for 2006 - 2007* | |
| Percutaneous (transrenal) extraction of a kidney stone | 5-550.2 |
| Percutaneous (transrenal) extraction of a kidney stone with lithotripsy | 5-550.3 |
| *Codes for 2008 - 2019* | |
| Percutaneous (transrenal) extraction of a kidney stone | 5-550.20 |
| Percutaneous (transrenal) extraction of a kidney stone with lithotripsy | 5-550.30 |
| **Open stone surgery** | |
| Nephrotomy | 5-551.1 |
| Pyelotomy | 5-551.2 |
| Pyelocalicotomy | 5-551.4 |
